# Ultra-Wideband and Wide-Angle Perfect Solar Energy Absorber Based on Titanium and Silicon Dioxide Colloidal Nanoarray Structure

**DOI:** 10.3390/nano11082040

**Published:** 2021-08-10

**Authors:** Pinghui Wu, Kaihua Wei, Danyang Xu, Musheng Chen, Yongxi Zeng, Ronghua Jian

**Affiliations:** 1 Fujian Provincial Key Laboratory for Advanced Micro-Nano Photonics Technology and Devices, Quanzhou Normal University, Quanzhou 362000, China; phwu@zju.edu.cn (P.W.); tcms@qztc.edu.cn (M.C.); clmzyx@163.com (Y.Z.); 2 School of Automation, Hangzhou Dianzi University, Hangzhou 310018, China; weikaihua@hdu.edu.cn; 3 College of Science, Zhejiang University of Technology, Hangzhou 310023, China; xudanyang@zjut.edu.cn; 4 School of Science, Huzhou University, Huzhou 313000, China

**Keywords:** solar energy absorber, ultra-broadband perfect absorption, silica colloidal nanoarrays, refractory metal, surface plasmon resonance

## Abstract

In this paper, we designed an ultra-wideband solar energy absorber and approved it numerically by the finite-difference time-domain simulation. The designed solar energy absorber can achieve a high absorption of more than 90% of light in a continuous 3.506 μm (0.596 μm–4.102 μm) wavelength range. The basic structure of the absorber is based on silicon dioxide colloidal crystal and Ti. Since the materials have a high melting point, the designed solar energy absorber can work normally under high temperature, and the structure of this solar energy absorber is simpler than most solar energy absorbers fabricated with traditional metal. In the entire wavelength band researched, the average absorption of the colloidal crystal-based solar energy absorber is as high as 94.3%, demonstrating an excellent performance under the incidence light of AM 1.5 solar spectrum. In the meantime, the absorption spectrum of the solar energy absorber is insensitive to the polarization of light. In comparison to other similar structures, our designed solar energy absorber has various advantages, such as its high absorption in a wide spectrum range and that it is low cost and easy to make.

## 1. Introduction

The energy crisis is an important factor that restricts social development. Developing a collection and utilization approach of renewable energy is a major method to solve the problem. Solar energy, as one of the most important clean and renewable energies, could become an inexhaustible resource of energy that could further reduce the use of traditional fossil fuel to keep our environment clean. As a result, efficient solar energy absorbers that can capture and convert light into electricity are in high demand. In recent years, many meta-materials have been made to absorb solar energy, and various perfect solar energy absorbers that can match solar radiation in broadband have been reported [1,2,3,4]. However, noble metals are generally employed in these solar energy absorbers, which will result in high cost. Solar energy absorbers, which can simultaneously satisfy the demand of utilizing solar as much as possible and are at a low cost, still need to be developed [5,6,7,8]. Therefore, we have tried to design a solar energy absorber that can satisfy all these requirements.

Since Landy et al.’s important report on the electromagnetic wave solar energy absorber in 2008, the electromagnetic wave solar energy absorber started to develop quickly. Many theories and experiments have shown that light-excited metal surface plasmon resonance can be achieved based on the metal–insulator–metal (MIM) structure, so it is possible to develop a perfect solar absorber [9,10,11,12,13,14]. However, because of the inherent single resonance of plasmonic nanostructure and meta-materials, most solar energy absorbers based on MIM structure only realize high absorption in a narrow spectrum band, not satisfying the requirement of the solar energy absorber [15,16,17]. Even if some solar energy absorbers can achieve broad-band absorption, their structures are usually very complex [15,18,19]. In addition, there is another colloidal crystal based on metamaterials. Studies have shown that by coupling solar radiation to the whispering gallery mode in the microspheres, the absorption efficiency of solar cells can be significantly improved [20,21]. Under the action of colloidal cavity and metal photon mode and surface plasmon, several narrow absorption peaks can usually be obtained in the absorption spectrum of the absorber [22]. Therefore, we can try to form a new resonance mode from the spherical nanostructure to capture light in the broadband range [23,24,25]. The dielectric microsphere-based solar energy absorber developed by Amir Ghobadi et al. can realize an ultra-wideband absorption in the wavelength range of 400–1000 nm [26]. Different from metal nanostructure, the photon dielectric cavity of the new mode significantly contributed to optics resonance and light absorption.

Titanium (Ti) is one of the metal materials commonly used in solar energy absorbers [27]. Thanks to the dielectric property, Ti possesses a strong plasmon resonance performance and has broad-spectrum absorption characteristics [28,29,30]. Additionally, the reserve of Ti is more abundant than traditional noble metals such as gold and silver in the natural world, meaning a low cost. Ti has good performance even under alkaline and acidic conditions. Additionally, as one of the refractory metals, Ti can work normally at high temperatures. At present, most of the light absorption of silicon dioxide is concentrated as a substrate material, ignoring many significant advantages of silicon dioxide as a surface structure. Studies have shown that semiconductors, such as silicon dioxide, can be used for plasmon coupling and absorption, and semiconductor resonators with strong optical coupling have been developed [31,32]. Meanwhile, the silicon dioxide structure can be made by the self-assembly method and does not need high technology processes such as electron beam lithography [33]. In this way, the colloidal crystal is manufactured extensively under a lower budget and time. In short, compared to the other materials commonly used in solar absorbers, titanium and silicon dioxide have obvious advantages as materials for solar absorbers.

Here, we propose a broad-spectrum solar absorber based on colloidal silica crystal array and titanium, which can achieve high absorption of solar energy over the longest possible wavelength band. As incident light can well resonate with a solar energy absorber, the designed solar energy absorber can achieve ultra-broadband perfect absorption in near-infrared to near-ultraviolet region. The designed solar energy absorber has an ultra-high absorption of more than 90% in the wavelength range of 0.596–4.102 μm, achieving high absorption that is higher than 80% in the wavelength range from 0.2 μm to 4.171 μm. In the entire region (0.2–4.2 μm), the average absorption is as high as 94.3%. The designed absorber is insensitive to the polarization of light and has good performance under a certain incident angle. It can also work for long-life time because the solar energy absorber is composed of refractory materials.

## 2. Structural Design of Broadband Perfect Solar Energy Absorber

**Figure 1:**
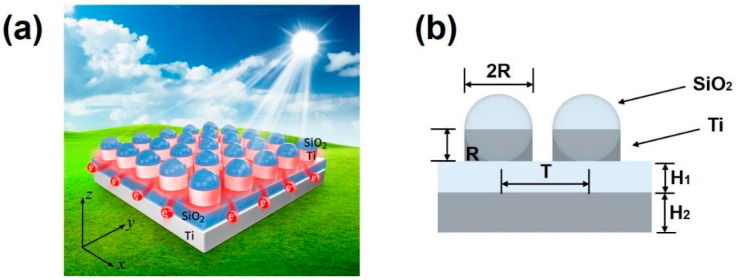
(**a**) Schematic diagram showing the ultra-broadband solar energy absorber. (**b**) The front view of the absorber.

The designed ultra-broadband solar energy absorber is shown in Figure 1a. Its substrate is composed of Ti and silicon dioxide. The surface microstructure consists of silicon dioxide colloidal crystal array and Ti. The thickness of SiO_2_ and Ti at the bottom is H_1_ = 0.145 μm and H_2_ = 0.450 μm, respectively. The radius of the surface silicon dioxide sphere is R = 0.25 μm, and the sphere is tangent to the SiO_2_ layer. The filling material between the sphere and SiO_2_ layer is Ti, whose height and radius are identical to the radius of the sphere. The front view is illustrated in Figure 1b, each cell of the microstructure includes a SiO_2_ sphere and filled metal. The unit cycle of the solar energy absorber is T = 0.6 μm.

The designed absorber was analyzed by using finite-difference time-domain (FDTD) simulation [34]. We use the commercial software FDTD solutions to simulate the model. A plane wave with a wavelength from 0.2 μm to 4.2 μm was used to illuminate from above in the Z-axis direction, and the boundary conditions in the Z-axis direction outside the structure area were set to a perfectly matched layer (PML) boundary condition. The periodic boundary conditions were employed in X-axis and Y-axis directions to reduce the time and resource during calculation. The override X mesh and Y mesh are set to 0.01 μm, and the override Z mesh is set to 0.005 μm, which is high precision. The refractive index data of SiO_2_ and Ti that are employed by the model are from Palik [35]. The transmitted light spectrum is detected at the bottom of the absorber in the Z direction, and a detector is set above the light source in the Z direction to detect the reflection spectrum.

## 3. Results and Discussion

**Figure 2:**
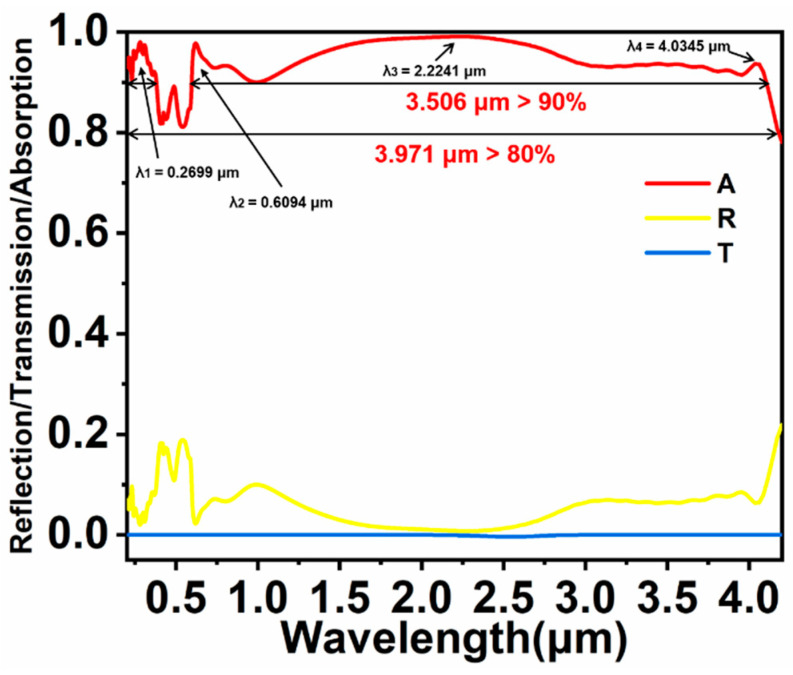
Optical spectra of the colloidal crystal-array-based solar energy absorber.

Figure 2 is the optical spectra of colloidal crystal-array-based solar energy absorber. A, R, and T represent the absorption, reflection, and transmission, respectively. Since the substrate used is thick enough to prevent the transmission of incident light, the T is closed to 0. Therefore, A is calculated through the formula A = 1 − R. In Figure 2, we can clearly observe that an absorption rate of > 90% spans 3.506 μm, and the absorption band reaches 3.971 μm (80% absorption rate). There are several distinct absorption peaks in the entire absorption band, such as λ_1_ = 0.2699 μm with an absorption rate of 97.55%, λ_2_ = 0.6094 μm (96.55%), λ_3_ = 2.2241 μm (99.12%), and λ_4_ = 4.0345 μm (99.74%). According to Figure 2, it is easy to obtain that the colloidal crystal-array-based solar energy absorber has excellent performance in the absorption of light, and the absorption band is wider than previous meta-material-based absorbers (Table 1) [36,37,38,39,40].

**Figure 3:**
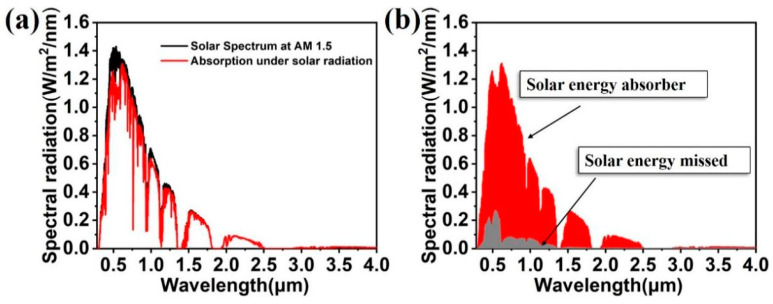
(**a**) Absorption spectrum of colloidal crystal-array-based solar energy absorber under AM 1.5 light source. (**b**) Solar energy absorbed and missed for colloidal crystal-array-based solar energy absorber under AM 1.5 light source.

The designed colloidal crystal-array-based solar energy absorber is applied to the broadband absorption of solar energy. The solar energy absorber was set under the ideal spectrum of AM 1.5. Figure 3a shows the absorption spectrum of the colloidal crystal-array-based solar energy absorber under an AM 1.5 light source. The solar energy absorber achieves a high absorption in almost the whole spectral regime. According to Formula (1), the solar absorption rate was calculated to be 90.9%.

(1) $$\alpha =\frac{\int_{\lambda min}^{\lambda max}\left(1-R\left(\omega \right)\right)\cdot AM1.5\left(\omega \right)d\omega }{\int_{\lambda min}^{\lambda max}AM1.5\left(\omega \right)d\omega }$$

Figure 3b shows the absorbed and missed solar energy for the solar energy absorber under the ideal spectrum. According to Formula (2), the solar energy loss rate was calculated to be 9.1%. Although the colloidal crystal-array-based solar energy absorber misses some solar energy, it will not affect the advantages of the solar energy absorber in the near-infrared and near-ultraviolet region. On the basis of Kirchhoff’s Law, the absorption rate for the object lacks an equal numerical value. In other words, the higher the absorption capacity of the object is, the greater the radiation capability of the object is. As a result, it is inferred that the designed solar energy absorber is qualified for the job of absorbing solar energy and exhibits potential to be utilized in more related devices such as a solar thermal generator and a heat transfer system [41,42].

(2) $$\beta =\frac{\int_{\lambda min}^{\lambda max}R\left(\omega \right)\cdot AM1.5\left(\omega \right)d\omega }{\int_{\lambda min}^{\lambda max}AM1.5\left(\omega \right)d\omega }$$

**Figure 4:**
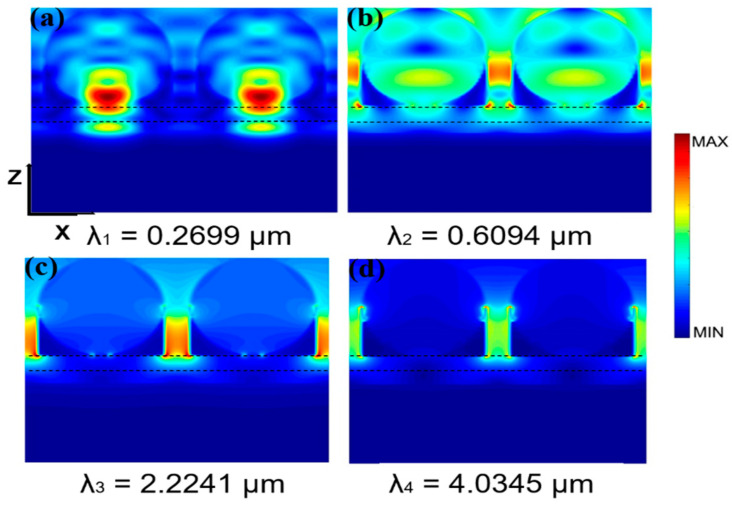
Electric field intensity distribution profiles on the X–Z plane under the excitation of incident light with wavelength of λ_1_ (**a**), λ_2_ (**b**), λ_3_ (**c**), and λ_4_ (**d**), respectively.

Figure 4 shows the electric field intensity distribution profiles of the colloidal crystal-array-based solar energy absorber at the four absorption peaks. In Figure 4a, field distribution is restricted to the region between spherical cavity and substrate, indicating that the spherical cavity can be resonantly coupled with the substrate. Meanwhile, there is strong field distribution in the substrate of Ti, showing that the substrate Ti not only impedes the transmission of light but also resonates with incident light. As shown in Figure 4b, the electric field is distributed in the spherical cavity and its surface, demonstrating that the solar energy absorber is resonantly coupled with the photon-guided mode of the spherical cavity. The field distribution also exists in silicon dioxide, which further explains that the substrate contributes to the improved absorption performance of the solar energy absorber [43,44]. The field distributions in Figure 4c,d are similar. The field is distributed between adjacent cells and exists between the filled Ti and silicon dioxide layer [39,45]. This is because the strong near-field coupling effect usually occurs between adjacent resonators or other plasmon resonances [46,47]. The difference is that there is also a strong plasmon coupling between the substrate and surface structure in Figure 4c [48,49]. Through these electric field intensity distribution profiles at absorption peaks, we have a good knowledge of the resonance coupling modes of colloidal crystal-array-based solar energy absorber.

**Figure 5:**
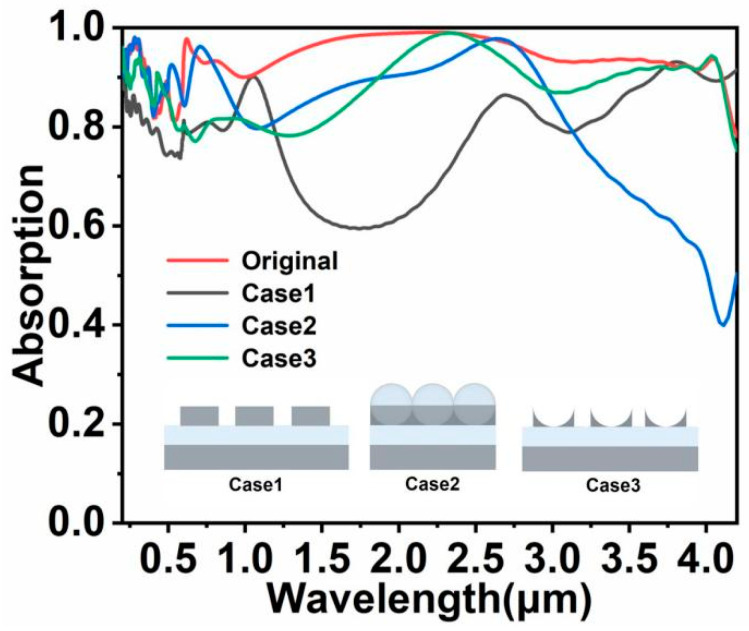
Absorption spectra of the original solar energy absorber, Case 1, Case 2, and Case 3, respectively.

To explore the effect of surface structure on the absorption spectrum of the solar energy absorber and the role of surface silicon dioxide spheres, solar energy absorbers with different structures were designed and simulated. Figure 5 shows the absorption spectra of original solar energy absorber, Case 1, Case 2, and Case 3, respectively. In Case 1, only the Ti cylinder is reserved in microstructure, and its thickness, period, and radius are not changed. In this case, the absorption spectrum of solar energy absorber possesses several discrete absorption peaks. The absorption spectrum is divided into two distinct regions by the lowest point of absorption at a wavelength of 1.68 μm. Over the entire wavelength range, the average absorption of Case 1 is only 80.78%. Most solar energy absorbers with MIM structure are simple, and are similar to Case 1, only achieving several narrow high absorption peaks on the absorption spectrum. Although Case 3 is also a MIM structure, the performance of Case 3 is better than Case 1—i.e., the average absorption of Case 3 is 87.48%. Compared to the original structure, the absorption of Case 1 and Case 3 is much lower, especially around the mid-wavelength band. It demonstrates that the silicon dioxide sphere has a huge influence on the absorption of the colloidal crystal-array-based solar energy absorber around the mid-wavelength band. In Case 2, the colloidal crystal array on the surface is in contact with each other. Under this condition, absorption of the solar energy absorber drops significantly in the long-wavelength band, and the average absorption of the solar energy absorber is 90.48% in the whole band, indicating that the space in between the colloidal crystal arrays is very important for plasmon coupling.

**Figure 6:**
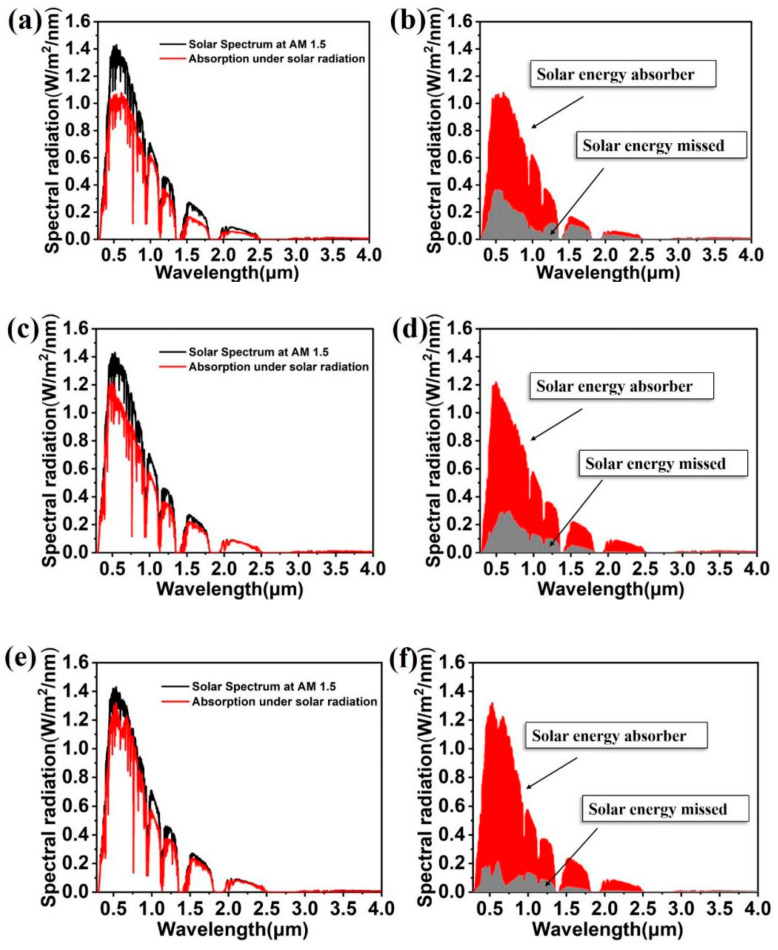
(**a**,**c**,**e**) Absorption spectrum of Case 1, Case 2 and Case 3 under AM 1.5 light source, respectively. (**b**,**d**,**f**) Corresponding solar energy absorbed and missed in Case 1, Case 2 and Case 3.

To further illustrate the advantages of the colloidal crystal-array-based solar energy absorber, the absorption spectra, i.e., the solar energy absorbed and missed in Case 1, Case 2, and Case 3 under AM 1.5 light source, were calculated and compared. Figure 6a–b, Figure 6c–d, and Figure 6e–f are the absorption spectra, the absorbed energy and missed energy diagram for Case 1, Case 2, and Case 3 under the AM 1.5 solar spectrum, respectively. The solar energy loss rates in these three cases are calculated by Formula (2) to be 19.54%, 16.56% and 10.01%, respectively. We can see that the strongest part of the AM 1.5 solar spectrum is concentrated on the visible to near-infrared light. Therefore, solar energy absorbers should possess a high absorption rate to visible and near-infrared light. However, when the solar energy absorber of Case 1 and Case 2 is put under the AM 1.5 solar spectrum, they lose much energy of the visible and near-infrared light. This will affect the absorption capacity of the solar energy absorber, especially when the solar energy absorber is applied in devices [50,51,52]. Although the average absorption of Case 3 is close to that of the original solar energy absorber, the average absorption is not the only standard to assess the performance of solar energy absorber. The wasted energy of Case 3 is much higher than the original solar energy absorber’s in the visible and near-infrared region. These demonstrate that the designed solar energy absorber has higher application potential than solar energy absorbers of similar structure.

**Figure 7:**
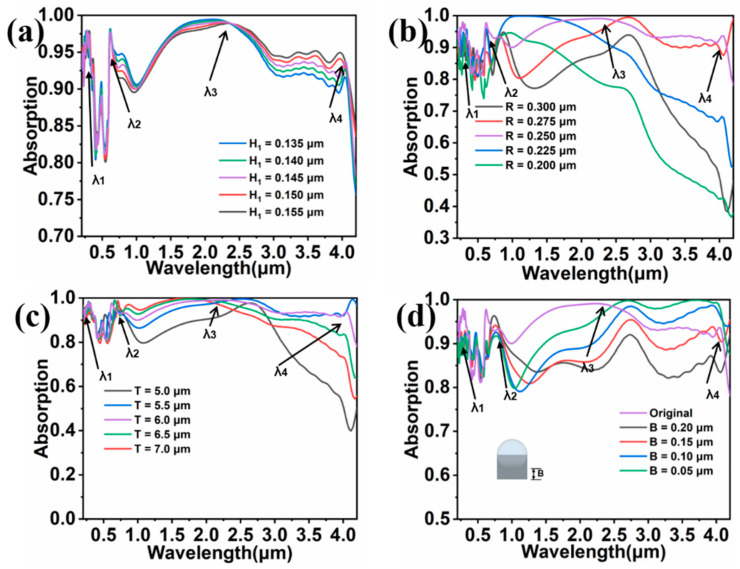
Evolution of absorption spectrum with structural parameter of (**a**) H_1_, (**b**) R, (**c**) T, and (**d**) B.

As the absorption property of solar energy absorber is often highly dependent on its structural parameters, various parameters of the colloidal crystal-array-based solar energy absorber were varied so as to investigate the effect on the absorption spectrum. Figure 7a shows the absorption spectra of solar energy absorber with different H_1_. As the thickness of H_1_ is increased, the absorption is gradually decreased near λ_2_ and gradually increased near λ_4_. This is because the silicon dioxide layer resonates with exciting light, and the resonance intensity in certain bands is changed with the variation of SiO_2_ thickness. As a result, the absorption spectrum is altered. In Figure 7b, the radius of the SiO_2_ sphere was changed. The change in the absorption spectrum in Figure 7b is not identical to that as shown in Figure 7a, because the surface structure involves more than one resonance mode. According to the results obtained in Figure 4, there is a strong near-field coupling effect and strong resonance coupling between surface structure and substrate near mid-wavelength band. When the radius of the SiO_2_ sphere is changed, two resonance modes are varied simultaneously, making the absorption spectrum change significantly. Additionally, we can clearly see that the parameter of the designed colloidal crystal-array-based solar energy absorber is very appropriate. In Figure 7c, we change the period of the solar energy absorber, namely, we change the distance of the adjacent surface microstructure. As T becomes larger, the absorption in the medium- and short-wavelength bands in between λ_2_ and λ_3_ is enhanced, but the absorption in the long-wavelength bands near λ_4_ is decreased. When the period T was varied from 5 μm to 5.5 μm, a large increase in the absorption is observed, meaning a critical point of coupling exists between T = 5 μm and T = 5.5 μm. Although the solar energy absorber with T = 5.5 μm has a high absorption in the long-wavelength band, it loses much energy in the mid-wavelength band where the solar energy is concentrated. Therefore, we comprehensively believe that the solar energy absorber with a period T of 6.0 μm is more suitable for a solar energy absorber. In Figure 4d, we attempt to explore the effect of distance B between SiO_2_ sphere and substrate. As the B is increased, the absorption with a wavelength larger than λ_2_ decreases gradually, and the absorption between λ_2_ and λ_3_ is lower than the absorption of the original solar energy absorber. According to Figure 7a–d, we reveal that the change of parameters of solar energy absorber has less effect on the short-wavelength band of the absorption spectrum. The reason is probably ascribed to the fact that the field distribution around λ_1_ is mainly restricted between the spherical cavity and substrate, and this resonance mode is less affected by structural parameters. In summary, the selected parameters of the colloidal crystal-array-based solar energy absorber are highly suitable.

**Figure 8:**
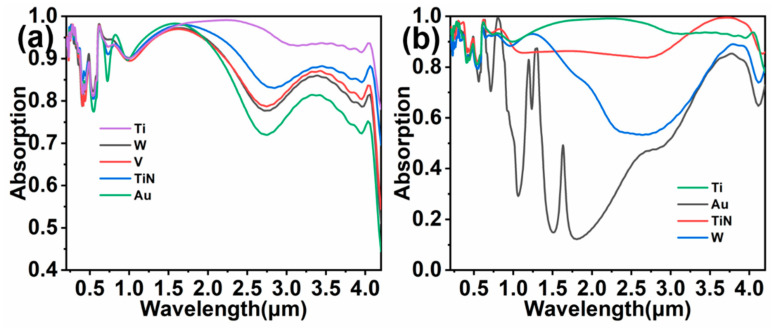
Absorption spectra of solar energy absorber with different substrates (**a**) and different filling materials in between the sphere cavity and substrate (**b**), respectively.

To investigate the influence of different metallic materials on the absorption spectrum of the colloidal crystal-array-based solar energy absorber, the materials of the solar energy absorber were changed. In Figure 8a, the Ti substrate is replaced with W, V, TiN, and Au substrate, respectively. The absorption spectrum almost exhibits a similar trend. The solar energy absorber with W, V, TiN and Au substrate has an absorption rate of 87.38%, 87.78%, 90.18%, 84.88%, respectively. Refractory metals have different imaginary parts, but their imaginary parts are generally higher, so the loss of light is higher. The difference between the absorption of the absorber using the Ti substrate and using other materials is mainly concentrated in medium- and long-wavelength bands, and the absorption of the solar energy absorber by using Au as the substrate is much lower than others obviously. This is because refractory metals, such as W, V, or TiN, as a substrate will resonate with incident light, which has been demonstrated in Figure 4. Au is a noble metal with high reflectivity, only hindering the transmission of light in the solar energy absorber. In Figure 8b, the filling materials in between the sphere and substrate are varied to Au, TiN, and W. The evolution of absorption in Figure 8b is similar to that shown in Figure 8a. The high reflectivity of Au gives rise to a significant decrease in the absorption of solar energy, and the solar energy absorber using Ti as filling materials has optimal performance. Overall, titanium is the most suitable material in this structure.

**Figure 9:**
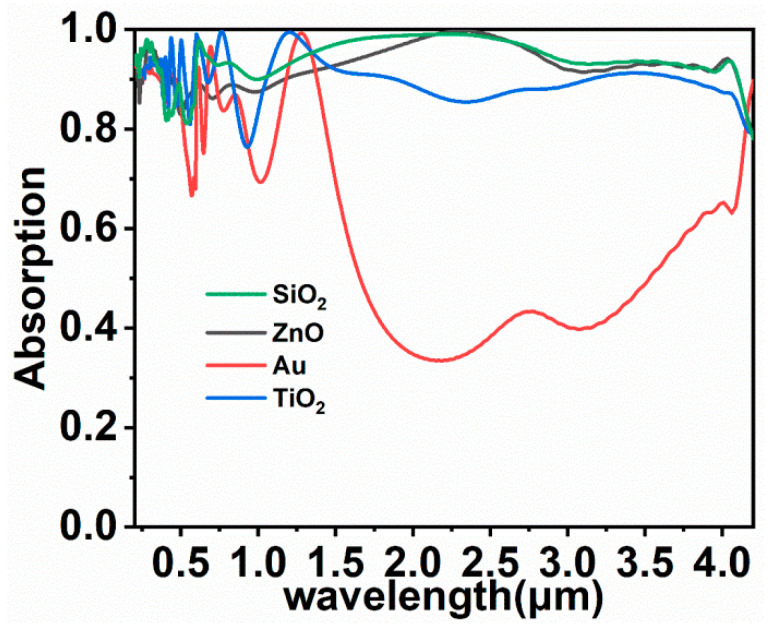
Absorption spectra of solar energy absorbers by employing different sphere materials.

Moreover, the material nanosphere was varied to study its effect. ZnO and TiO_2_ are also commonly used nanosphere materials in solar energy absorbers [53,54]. In Figure 9, the material of the nanosphere is replaced with ZnO, TiO_2_, or Au, and their average absorption is 92.94%, 89.71% and 59.13%, respectively. We can see that both ZnO and TiO_2_ can realize good absorption. Therefore, it is believed that colloidal crystal-array-based solar energy absorbers have the potential to be extended for use in other materials and can maintain their performance in different optoelectronic devices [55], whereas high-reflective materials, such as Au, are not suitable as sphere materials.

**Figure 10:**
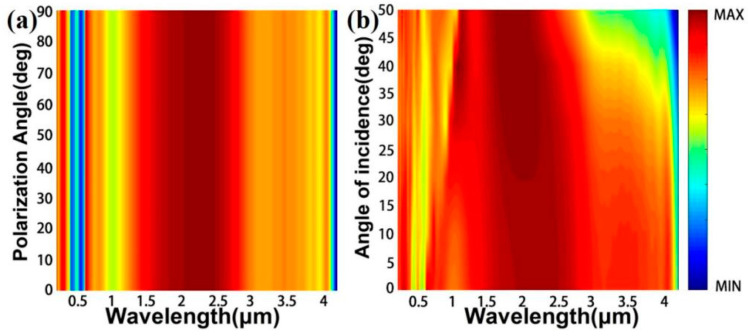
Absorption spectra of the designed solar energy absorbers under (**a**) different polarizations and (**b**) incident angles.

Normally, the direction of the incident light is not fixed in nature [56,57,58]. The absorption capacity under incident light with different angles is also an important factor for the assessment of the performance of the solar energy absorber. In Figure 10a, we put the colloidal crystal-array-based solar energy absorber under the TE and TE polarization light. In the entire researched band, the absorption of the designed solar energy absorber is not sensitive to incident light by changing the polarization from TE to TM. This is because the designed solar energy absorber adopts a highly symmetrical structure [59]. Figure 10b is the result of the solar energy absorber under incident light with different angles. We can clearly observe that when the incident light angle is smaller than 40°, the absorption of the solar energy absorber is slightly affected. A high absorption is present in the whole spectral region. When the incidence angle is larger than 40°, the change of the incident angle has a greater influence on the absorption of the solar energy absorber. In general, we believe that colloidal crystal-array-based solar energy absorbers can satisfy the requirements for solar energy absorbers in practical applications.

**Figure 11:**
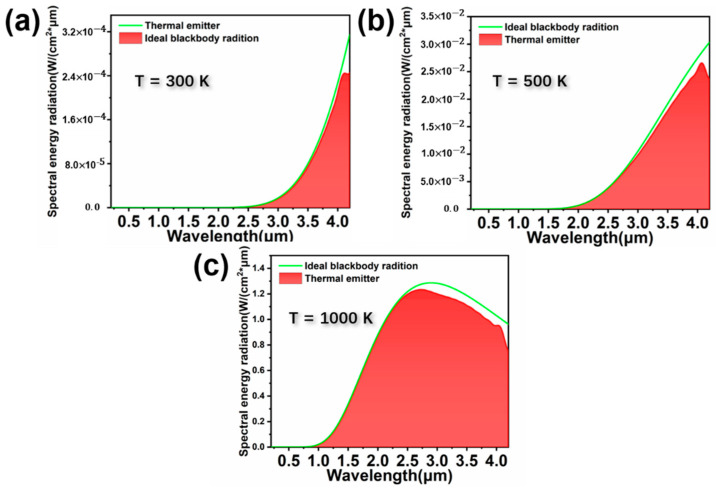
(**a**–**c**) are the heat radiation effect diagrams of the structure at 300 K, 500 K and 1500 K, respectively.

At the end, we analyzed the heat radiation of the designed absorber. As shown in Figure 11a–c, we have selected three representative heat radiation effect diagrams at different temperatures. The heat radiation efficiency (η_E_) is an important parameter for evaluating the thermal emission performance of solar absorbers, which is defined as follows [60]:

(3) $$\eta_{E}=\frac{\int_{\lambda_{min}}^{\lambda_{max}}\varepsilon \left(\omega \right)\cdot I_{BE}\left(\omega ,T\right)d\omega }{\int_{\lambda_{min}}^{\lambda_{max}}I_{BE}\left(\omega ,T\right)d\omega }$$

where, $\;I_{BE}\left(\omega ,T\right)$ is the spectral intensity of an ideal black body at a certain frequency *ω* and temperature *T*. It can be obtained that the $\eta_{E}$ of the designed absorber at *T* = 300 K, 500 K and 1000 K are 91.12%, 92.58% and 95.12%, respectively. The heat radiation efficiency increases as the temperature of the absorber increases. At the same time, since the material used in the designed absorber has a higher melting point, it can meet the requirements of practical applications.

## 4. Conclusions

In this work, we have designed an ultra-wideband solar energy absorber and performed simulations on it. The surface structure is made of silica colloidal crystals and filling titanium, and the substrate is composed of titanium and silicon dioxide, which act as electromagnetic wave coupling and trap resonators. This structure has the characteristics of ultra-high absorption of light in the visible to near-infrared range (0.596 μm–4.102 μm). We compared the influence of different metal materials and structural geometric parameters on the absorption performance and revealed the absorption physical mechanism of the absorber through the electric field diagram and the magnetic field diagram. The absorption capacity under the real solar spectrum was also simulated to evaluate the application potential of the solar energy absorber. The proposed absorber has the advantage of a simple structure, as well as ultra-wideband absorption that is insensitive to polarization. At the end, we also evaluated the heat radiation capacity of the absorber through its heat radiation efficiency. To conclude, the absorber proposed in this paper has broad prospects as regards light-to-heat conversion equipment, solar power generation and has perfect stealth.

## Figures and Tables

**Table 1:** Absorption range of different structures with an absorption rate of more than 90%.

Reference	Structure	Absorption Range	Wavelength Range With Absorption Rate More than 90%	Maximal/Average Absorption Rate
[36]	TiN and TiO_2_ disc	0.316–1.426 μm	1.11 μm	99.8%/93%
[37]	SiO_2_ and TiN disc	0.516–2.696 μm	2.18 μm	99%/~
[38]	SiO_2_ sphere and Ge	1.283–2.830 μm	1.547 μm	98%/88%
[39]	Metal–dielectric–metal	0.3–1.1 μm	0.8 μm	99.5%/91.6%
[40]	SiO_2_ and TiO_2_ cubes	0.405–1.505 μm	1.1 μm	99.9%/95.1%
This work	SiO_2_ sphere and Ti	0.596–4.102 μm	3.506 μm	99.74%/94.3%

## Data Availability

Publicly available datasets were analyzed in this study. This data can be found here: [https://www.lumerical.com/].
